# Surgery

**Published:** 1893-03

**Authors:** W. M. Barclay


### SURGERY.

The treatment of spinal abscesses is one of such importance that its discussion is always interesting, and Mr. Watson Cheyne did well to make it the subject of an address in the section of Surgery at the Nottingham meeting last year.[1] The introduction of the aseptic method was the first great advance in the treatment, and in the hands of Lister was very successful. Thus of 37 patients treated by him in Edinburgh, between 1871 and 1879, by simple aseptic drainage, 23 (or 62.1 per cent.) were certainly cured, although the percentage of probable cures was as high as 72.9;[2] and, if the last four years only are considered, the number of certain cures amounted to 73.3 per cent.[3] Taking into consideration the usual course of these cases, and the inevitable percentage of mortality from secondary infection or wide extension of the disease, these results were brilliant. In the last few years, however, much very valuable work has been done in elucidating and establishing the pathology of the tubercular process, one effect of which has been to open out the whole subject of treatment once more.

In the first place, it is well known that the so-called "tubercular abscess" can, under favourable circumstances, become absorbed. What are the chances of such an event in the case of a spinal abscess? Dr. Burrell[4] quotes a case where "a large fluctuating tumour" in the thigh connected with Pott's disease disappeared, and states that he could cite several other instances of a similar nature. He concludes "that under an expectant plan of treatment the abscess will, in many cases, disappear." Dr. Townsend, another American observer, gives a statistical examination of 380 cases of uncomplicated cases of

[1] *Brit. Med. Journ.*, Dec. 31, 1892.

[2] *Antiseptic Surgery*, 1882, p. 536. [3] *Op. cit.*, p, 538.

[4] *Med. News*, Dec. 12, 1891.

Pott's disease "in all stages," of which 75 were complicated with abscess.[1] Of these 75 cases, 21 received "no treatment but the brace," and of these 21 the abscess disappeared in 3 only, remaining stationary in 8, and increasing in 10. Mr. Cheyne, in his address, quotes Lacharrière as having found a record of 44 cases of spontaneous absorption. Yet probably few surgeons would deny that such a possibility is only to be taken into consideration when the patient's general condition precludes any more active treatment. According to Mr. Howard Marsh, "this event is so rare that it ought not to be allowed to interfere with any general rule of practice."[2]

In the second place, there is the question of aspiration as a means of bringing about the same result more quickly. While Dr. Burrell considers it "an uncertain measure," Dr. Townsend reports 11 successful aspirations out of 19.

Finally, we come to the latest development of the aseptic method—a method which is able to give a final cure in 19 out of 21 cases, deducting 3 deaths, of which only 1 can be attributed to the treatment employed.[3] Recognising, as we now do, that a "tubercular abscess" is really a mass of tubercular deposit which has disintegrated in the centre—the still solid peripheral portion forming the "wall" of the abscess—any treatment which leaves this "wall" untouched is manifestly imperfect; and it is to remedy this defect that all recent efforts are directed. This method consists essentially in evacuating the "pus," flushing the cavity with a hot fluid, and scraping away as far as possible all the tubercular "wall," removing at the same time any sequestra that may be found, or scraping a carious patch. As early as 1884 Mr. Treves urged the evacuation of these abscesses through an incision in the loin, as allowing of direct access to the focus of disease in the vertebræ, and of removal of the pus and *débris* by the shortest route—a method which has recommended itself to the majority of surgeons. For flushing the cavity Mr. Barker employs sterilised water at 105°—110° F., Mr. Treves prefers a 1 in 5000 solution of corrosive sublimate at 100° F., and Mr. Cheyne a solution of only half this strength. For scraping the "wall," Mr. Treves recommends the finger as the safest instrument wherever it can reach, but it will generally have to be aided by some scraping instrument to reach the remoter parts and branching diverticula—or "a piece of fine Turkey sponge held in a slender long-bladed holder" can be employed, "with which the wall should be literally scrubbed."[4] Mr. Barker has adapted his "flushing gouge," which he first invented and described for the treatment of abscesses connected with hip disease,[5] to these cases by having them made of varying lengths.[6] When the flushing

[1] *Med. News*, Dec. 19, 1891. [2] *Brit. Med. Journ.*, Aug. 3, 1892.
[3] *Ibid.*, Dec. 31, 1892.
[4] *Manual of Operative Surgery*, 1891, vol. ii., p. 731 *et seq.*
[5] *Brit. Med. Journ.*, Jan. 19, 1889. [6] *Ibid.*, Feb. 7, 1891.

fluid returns clear, the cavity is wiped dry and the wound closed by deep sutures, including the muscular structures, without, as a rule, the employment of any drainage. Mr. Barker pours in 2—3 ounces of iodoform emulsion before closing the wound, any excess being squeezed out before tying the sutures [1]—the object being to prevent the development of any tubercle bacilli that may still be left behind; and Mr. Cheyne concludes that "on the whole the iodoform does some good." It need scarcely be pointed out that these measures can only be successfully carried out with the most rigid antiseptic precautions. A case of Pott's disease which came under my care last year exemplifies the good results of this method. The patient (E. H., aged 21) had angular curvature (said to be of three months' duration) and a tense fluctuating swelling in the right iliac fossa. On August 30 I opened the psoas abscess in the loin, scraped the "wall" with finger and sharp spoon—irrigating constantly with 1 in 5000 perchloride solution,—gouging a small carious patch in one of the bodies, finally drying the cavity with pieces of sponge and pouring in a 10 per cent. iodoform emulsion. The only point in which I departed from the method as detailed above was in using a drainage-tube for the first few days. I am inclined to think that the use of a drainage-tube for the first twenty-four hours is an advantage in such cases to get rid of the first large serous oozing, and it cannot possibly interfere with the primary union we aim at. In the present case I left it in longer because I did not feel sure that some diverticulum had not escaped the sharp spoon. The wound healed soundly under an antiseptic dressing in six weeks. The patient was kept in the recumbent position till January 16 of this year, when, being obliged to go home, she was discharged with a Sayre's jacket on the 28th. The wound was sound, the flank and iliac fossa were resonant, and she was free from all pain or discomfort.

This method is most promising, and certainly rests on a sound basis.

The treatment of flat-foot has hitherto been chiefly directed to mechanical support of the depressed arch without much regard to the physiological functions of the foot. The wearing of boots with heels not too high, and "made to extend on the inner side to a point opposite the middle of the foot," and "fitted with a 'valgus pad' so as to support the natural arch of the foot," or, in more severe cases, the employment of Little's boot, [2] pretty nearly sums up the usual teaching in this matter. But a careful consideration of the construction and functions of the foot has led to a more rational line of treatment in these cases. Dr. Whitman, in a paper on this subject, [3] has defined

[1] *Loc. cit.*

[2] ERICHSEN, *Science and Art of Surgery*, ninth edition, 1888, vol. ii., p. 548.

[3] *New York Med. Journ.*, Feb. 27, 1892.

flat-foot as "an acquired partial dislocation, caused by a disproportion between the weight to be sustained and the strength of the supporting structures." Its causation may be usefully considered under three heads: (1) Improper walking or standing, (2) overweighting of the foot, (3) neglect of the universal law of intermittent action.

1st. According to Dr. Whitman[1] and Mr. Ellis,[2] the strong position of the arch is that of slight adduction (*i.e.* with the toes pointing slightly inwards), and extension of the foot (as in rising to tiptoe) can only take place in combination with adduction.[3] Anything, therefore, which interferes with these movements, whether corns, ill-fitting boots, or bad walking, so far tends towards this deformity.

2nd. Flat-foot is most common at the time of life when growth is rapid, and apt to be (especially in the poorly-nourished) out of all proportion to the ligamentous and muscular development—the arch sinking under a burden greater than it can bear.

3rd. "The foot is supported not only *in*, but also *by*, the exercise of its functions. The muscles, which by action move, in action sustain the structure." "Ligaments are insufficient to resist tension when continuous or prolonged. On the other hand, intermittent tension promotes their strength."[4] The arch is maintained, during standing, mainly by means of its ligaments, but, in the act of walking, the very muscles and tendons that effect the movements brace up the arch and relieve the strain on its ligamentous supports. If then the foot is subjected to a too long-continued strain in either direction—standing or walking—the ligaments become relaxed, and the muscles crippled and paralysed, so that the normal balance of the foot is destroyed. In fully-developed painful flat-foot the power of adduction is lost, inflammatory adhesions have formed between the bones, and there is a contraction and shortening of the ligaments on the outer side, and a relaxation and lengthening of those on the inner side of the foot, so that passive movements cannot, as in mild cases, restore the shape of the foot. In a recent paper[5] Dr. Whitman enforces these conclusions, and tabulates 24 cases (of the severe type) successfully treated. The method he advocates is as follows: The foot is forcibly moved in every direction, to break up adhesions and overstretch all contracted parts, and finally extended and twisted inwards so as to *overcorrect* the deformity. A well-fitting plaster bandage is then applied in the overcorrected position. This is generally worn for about three weeks, by which time, he says, all inflammatory appearances have usually disappeared, and the patient

[1] *Loc. cit.* [2] *The Human Foot*, 1889, p. 24.

[3] *Cf.* QUAIN'S *Anatomy*, tenth edition, 1892, vol. ii., pt. ii., p. 198. HUMPHRY, *The Human Skeleton*, 1858, p. 568.

[4] ELLIS, "Nature and Treatment of Flat-foot," *Edin. Med. Journ.*, Jan., 1890.

[5] *Annals of Surgery*, vol. xvii., pt. i.

is then allowed to walk about on the foot wearing a light steel support or "brace."[1] Passive, followed by active, movements of extension and adduction are carried out daily, combined with massage and instruction in proper walking and standing; and this is continued till the voluntary control over these movements is completely restored. Mr. Howard Marsh has quoted[2] a similar procedure of Mr. Willett's; but in this method correction of the deformity is attempted in the *flexed* position of the foot. Mr. Ellis discards everything in the form of mechanical support, relying on due attention to the proper alternation of rest and activity, careful exercises and good walking, frequent rising to tiptoe during standing, and the use of boots which offer no hindrance to proper flexion of the foot (at the metatarso-phalangeal joint), or to the inward movement of the great toe in action. He contends that "good walking and many forms of work are not only compatible with the cure of flat-foot, but may be used as a direct influence to that end. They who walk well will never be flat-footed."[3]

The question of tranfusion is one which is sure from time to time to present itself in the course of surgical practice. It labours, however, under two very grave disadvantages, (1) the difficulty of obtaining blood, (2) the dangers (chiefly thrombosis) attending the process, which no care can avoid or foresee. Dr. William Hunter, in his lectures at the College of Surgeons,[4] treated this subject exhaustively. He showed that any value possessed by blood-transfusion over that of a neutral saline solution must depend on the red corpuscles, since he found that blood-serum has no properties that are not shared by a $\frac{3}{4}$ per cent. solution of common salt.[5] The implanted red corpuscles exert their oxygen-carrying function as long as they remain in the circulation, and the question is therefore narrowed to an inquiry into their fate. Dr. Hunter has shown that they are destroyed after a shorter or longer stay in the blood, the rapidity of this process depending directly on the activity of the metabolic processes at the time, and the quantity of blood transfused. The first factor is variable and uncertain, but the second is in man so limited that the life duration of the red discs is probably to be reckoned by hours only.[6] Dr. Hunter believes, however, that "there is scarcely a single condition of the blood in which the want of red corpuscles is a source of urgent danger."[7]

There are two conditions in which transfusion is chiefly called for:

(1) Acute traumatic anæmia, whether it be blood-formation that fails, or (by far the commonest event) failure of the

[1] For details of construction, etc., see *New York Med. Journ.*, Feb. 27, 1892.
[2] "Orthopædic Surgery," *St. Bartholomew's Hospital Reports*, vol. xviii., 1882.
[3] *Edin. Med. Journ.*, Jan., 1890.
[4] *Brit. Med. Journ.*, July 20, Aug. 3 and 10, 1889. [5] *Loc. cit.*, p. 119.
[6] *Loc. cit.*, p. 305. [7] *Loc. cit.*, p. 308.

circulation that is threatened. In the first alternative (theoretical considerations notwithstanding) experiments show that recovery "is effected with greater rapidity when simple saline solution is injected than when blood is transfused."[1] In the second the failure of circulation is due simply to the great diminution in volume of the blood causing such a fall in the blood-pressure that the circulation can no longer be maintained. Here the only indication is to restore the original volume as speedily as possible, and this a saline solution can effect as readily as blood.

(2) Shock. This state is in its essence an internal hemorrhage, inasmuch as the weak action of the heart and the loss of vascular tone accompanying it lead to the stagnation of a large portion of the blood in the venous system. Increasing the volume of the blood ought therefore to have the same effect here as in cases of actual hemorrhage.

Mr. R. J. Pye-Smith records[2] five cases where he employed intra-venous injection of saline fluid, with a successful result in three; and Dr. Sturges[3] reports a recovery from collapse following upon vomiting and diarrhœa under this treatment.

As to the method of performance: all are agreed that the simpler the apparatus the better, a glass cannula with a funnel and tube being quite efficient, and safer than any forcing apparatus. With regard to the quantity of blood to be injected and its temperature there are differences of opinion. The general impression seems to be that the temperature must not exceed that of the body, and that from 12 ounces to 1½ or 2 pints is a sufficient quantity of fluid. Dr. E. Sternberger has, however, just published[4] two cases in which he successfully injected three pints of a .6 per cent. solution of salt at temperatures of 117° and 118° F., in one case repeating the infusion the next day; and he mentions eight previous cases in which he had used this treatment, but only employing 1 to 1½ pints of fluid at the body temperature, unsuccessfully in all. He attributes his success in his last cases to the greater bulk and higher temperature of the solution, as well as to the administration of strychnia (gr. $\frac{1}{20}$ of the sulphate hypodermically) immediately after the infusion, and continued subsequently by mouth in one of the cases. Dr. Johnson-Alloway records[5] a case in which he practised peritoneal transfusion of three quarts of sterilised salt solution at a temperature of 110° F. for extreme collapse following the removal of the same quantity of peritonitic fluid. This had a most marked effect, although the abdomen in a few hours had become as flat as at the conclusion of the operation. In two or three of Mr. Pye-Smith's cases the injection was made

[1] *Loc. cit.*, p. 308.

[2] *Lancet*, 1892, vol. i., p. 913. [3] *Ibid.*, p. 86.

[4] *Med. Rec.*, Jan. 7, 1893.

[5] *Montreal Med. Journ.*, Feb., 1892.

previous to or in the course of an amputation, combating the shock and making the operation practicable. Saline injection has in such cases probably a large field of usefulness, and is well worthy of trial.

W. M. BARCLAY.

